# Early Use of Tocilizumab May Prevent Clinical Deterioration in Select COVID-19 Patients: A Case Series

**DOI:** 10.7759/cureus.9187

**Published:** 2020-07-14

**Authors:** Asif Hitawala, Sany Kumar, K V Gopalakrishna

**Affiliations:** 1 Internal Medicine, Cleveland Clinic Fairview Hospital, Cleveland, USA

**Keywords:** covid-19, severe acute respiratory syndrome coronavirus 2, tocilizumab, acute respiratory distress syndrome, interleukin-6, pandemic

## Abstract

Coronavirus disease 2019 (COVID-19) is a respiratory viral illness caused by the novel severe acute respiratory syndrome coronavirus 2. It is known to cause severe illness in certain patients, who develop acute respiratory distress syndrome (ARDS) often requiring intubation and mechanical ventilation adding to significant morbidity and mortality. Tocilizumab is an interleukin-6 inhibitor that has shown promise in improving outcomes in patients with COVID-19. It is usually administered to patients with severe COVID-19 who develop ARDS. We present three cases of COVID-19 where the patients were admitted to the hospital for observation and were found to be worsening clinically. They were believed to be developing ARDS, and intubation and mechanical ventilation were anticipated. Tocilizumab was administered in the early phase of the disease before intubation. Patients improved clinically and ultimately did not require intubation. Our findings suggest that early use of tocilizumab might be beneficial in preventing clinical deterioration and intubation in select COVID-19 patients.

## Introduction

The world is currently experiencing a coronavirus disease 2019 (COVID-19) pandemic caused by the novel severe acute respiratory syndrome coronavirus 2 (SARS-CoV-2). A recent Chinese report suggested that of the multitude of COVID-19 cases, about 14% are severe and 5% are critical [1]. These cases often require intubation and mechanical ventilation. Tocilizumab (TCZ) is an interleukin-6 (IL-6) inhibitor that has shown promise in reducing the severity of this illness with multiple ongoing trials (details can be found at www.clinicaltrials.gov). We present three cases of COVID-19 where the patients were worsening based on clinical as well as laboratory parameters and were given TCZ early in the disease course. They were believed to be heading towards the development of acute respiratory distress syndrome (ARDS) but ultimately did not require intubation and mechanical ventilation.

## Case presentation

Case 1

A 55-year-old male presented to the emergency room with complaints of worsening shortness of breath. He had contacted his physician 11 days ago complaining of low-grade fever and nasal congestion and was tested for COVID-19. On arrival to the emergency room, he was hemodynamically stable and his oxygen saturation (SpO_2_) was 93% on room air. Laboratory results were as shown in Table 1.

**Table 1:** Clinical and Laboratory Parameters of Case 1 Temp, maximum recorded oral temperature; HR, maximum recorded heart rate; RR, maximum recorded respiratory rate; FiO_2_%, fraction of inspired oxygen; WBC, serum white blood cell count; Abs Lym C, serum absolute lymphocyte count; Hb, serum hemoglobin; Hct, serum hematocrit; Plt count, serum platelet count; CRP, serum C-reactive protein; PT, prothrombin time; ALT, serum alanine aminotransferase; AST, serum aspartate aminotransferase; BUN, blood urea nitrogen; creat, serum creatinine; °F, degrees in Fahrenheit.

Parameters	Day 1	Day 2	Day 3	Day 4	Day 5	Day 6	Day 7
Temp (97.6°F-99.6°F)	98.6	99	98.4	98.1	98.1	98	97.8
HR (60-100/minute)	87	90	92	93	75	96	88
RR (12-20/minute)	23	24	30	24	16	18	16
FiO_2_ (%)	21	36	28	24	24	21	21
WBC (3.70-11 x 10^3^/µL)	5.80	-	3.56	10.61	-	7.28	-
Abs Lym C (1.00-4.00 x 10^3^/µL)	1.05	-	-	1.69	-	1.90	-
Hb (11.5-15.5 gm/dL)	14.2	-	13.9	13.5	-	14.6	-
Hct (36.0%-46.0%)	41.3	-	40.1	39.6	-	42.7	-
Plt count (150-400 x 10^3^/µL)	194	-	225	261	-	261	-
CRP (0.0-1.0 mg/dL)	10.1	9.7	4.4	28.2	1	1	0.5
Ferritin (30.3-565.7 ng/mL)	1,921	-	-	-	1,337	-	-
Fibrinogen (200-400 mg/dL)	717	-	-	-	420	-	-
Interleukin-6 (≤5 pg/mL)	<5	15	-	-	-	112	-
PT (0.9-1.3 seconds)	-	1.1	-	-	-	-	-
ALT (5-50 U/L)	65	82	183	313	-	185	-
AST (7-40 U/L)	53	49	81	123	-	38	-
BUN (9-24 mg/dL)	12	12	18	23	-	20	-
Creat (0.73-1.22 mg/dL)	0.81	0.88	0.73	0.85	-	0.96	-

**Figure 1:**
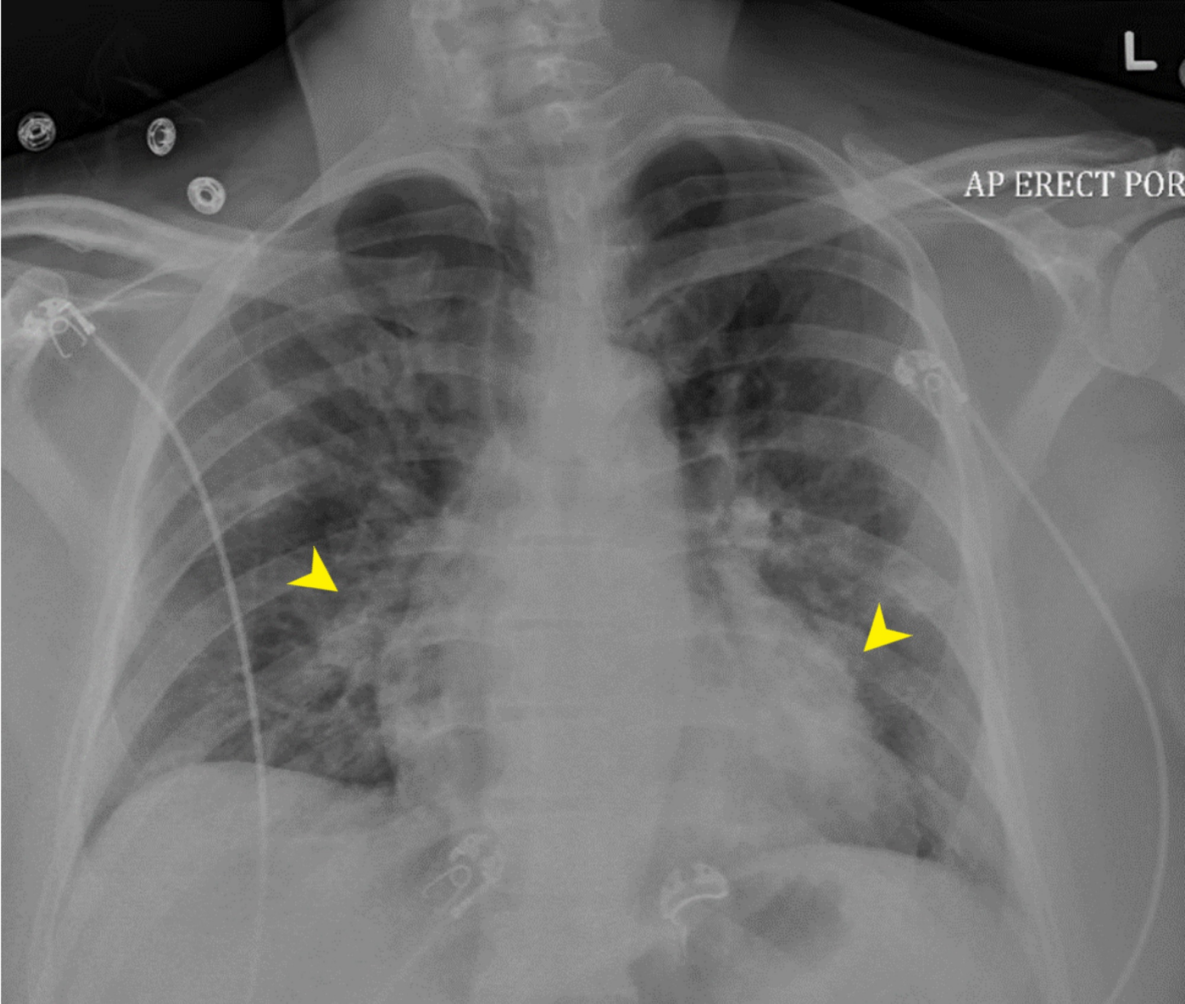
Chest X-ray shows patchy opacities especially in the left midlung region

Chest X-ray showed patchy opacities bilaterally (Figure 1).

He was started on hydroxychloroquine and azithromycin and admitted to the regular nursing floor for monitoring. Overnight, the patient started getting more hypoxic and was placed on three liters of oxygen via nasal cannula saturating about 94%. His hypoxia continued to get worse throughout the day and his inflammatory markers were uptrending (Table 1). He was given one dose of TCZ 400 milligrams (mg) and intravenous methylprednisolone 60 mg once on the second day of admission and transferred to the intensive care unit (ICU) in anticipation of intubation. He was on six liters of oxygen via nasal cannula and was noted to desaturate to 81%-85% while talking. He was observed overnight and the decision was taken to hold off on intubation. The patient started improving the next day, and his inflammatory markers started trending down. He was observed in the ICU for two days and then transferred to the regular nursing floor. His liver enzymes started uptrending, and hydroxychloroquine was stopped after three days. The patient, however, received five days of azithromycin. He was discharged on the sixth day of hospitalization with home oxygen. On a one-month follow-up, the patient has been doing well and his liver enzymes are back to normal.

Case 2

A 53-year-old male presented to the emergency room with complaints of shortness of breath. About 11 days ago, he started having fever, chills, fatigue, and generalized body aches. He was tested for COVID-19 six days before admission and was started on a five-day course of hydroxychloroquine and azithromycin which he completed. On arrival to the emergency room, he was found to be in mild respiratory distress and placed on five liters of oxygen via nasal cannula. He was otherwise hemodynamically stable. Laboratory results were as shown in Table 2.

**Table 2:** Clinical and Laboratory Parameters of Case 2 Temp, maximum recorded oral temperature; HR, maximum recorded heart rate; RR, maximum recorded respiratory rate; FiO_2_%= fraction of inspired oxygen; P/F ratio, ratio of arterial oxygen partial pressure (PaO_2_ in mmHg) to fractional inspired oxygen (expressed as percentage); WBC, serum white blood cell count; Abs Lym C, serum absolute lymphocyte count; Hb, serum hemoglobin; Hct, serum hematocrit; Plt count, serum platelet count; CRP, serum C-reactive protein; PT, prothrombin time; ALT, serum alanine aminotransferase; AST, serum aspartate aminotransferase; BUN, blood urea nitrogen; creat, serum creatinine; °F, degrees in Fahrenheit.

Parameters	Day 1	Day 2	Day 3	Day 4	Day 5	Day 6	Day 7
Temp (97.6°F-99.6°F)	99.5	99	98.8	98.4	97.9	97.8	97.9
HR (60-100/minute)	106	98	98	83	79	99	107
RR (12-20/minute)	26	33	24	21	20	20	22
FiO_2_ (%)	40	40	40	40	40	32	34
P/F ratio	268	165	-	-	-	-	-
WBC (3.70-11 x 10^3^/µL)	7.75	8.02	9.96	4.95	4.83	-	-
Abs Lym C (1.00-4.00 x 10^3^/µL)	0.77	-	1.37	1.35	1.59	-	-
Hb (11.5-15.5 gm/dL)	15.1	14.2	13.7	14.7	14.4	-	-
Hct (36.0%-46.0%)	43.6	41.5	40.9	42.8	43.0	-	-
Plt count (150-400 x 10^3^/µL)	255	290	297	184	275	-	-
CRP (0.0-1.0 mg/dL)	16.8	13.3	5.5	2.6	1.5	0.9	0.5
Ferritin (30.3-565.7 ng/mL)	1,770	1,849	1,287	983.6	1,061	1,050	1,076
D-dimer (<500 ng/mL)	-	3,030	-	-	-	-	-
Fibrinogen (200-400 mg/dL)	-	621	483	392	337	278	225
Interleukin-6 (≤5 pg/mL)	-	<5	-	-	-	-	-
PT (0.9-1.3 seconds)	-	1.1	-	-	-	-	-
ALT (5-50 U/L)	60	53	43	46	83	-	-
AST (7-40 U/L)	79	51	32	31	59	-	-
BUN (9-24 mg/dL)	7	11	18	16	14	-	-
Creat (0.73-1.22 mg/dL)	0.82	0.74	0.76	0.77	0.75	-	-

**Figure 2:**
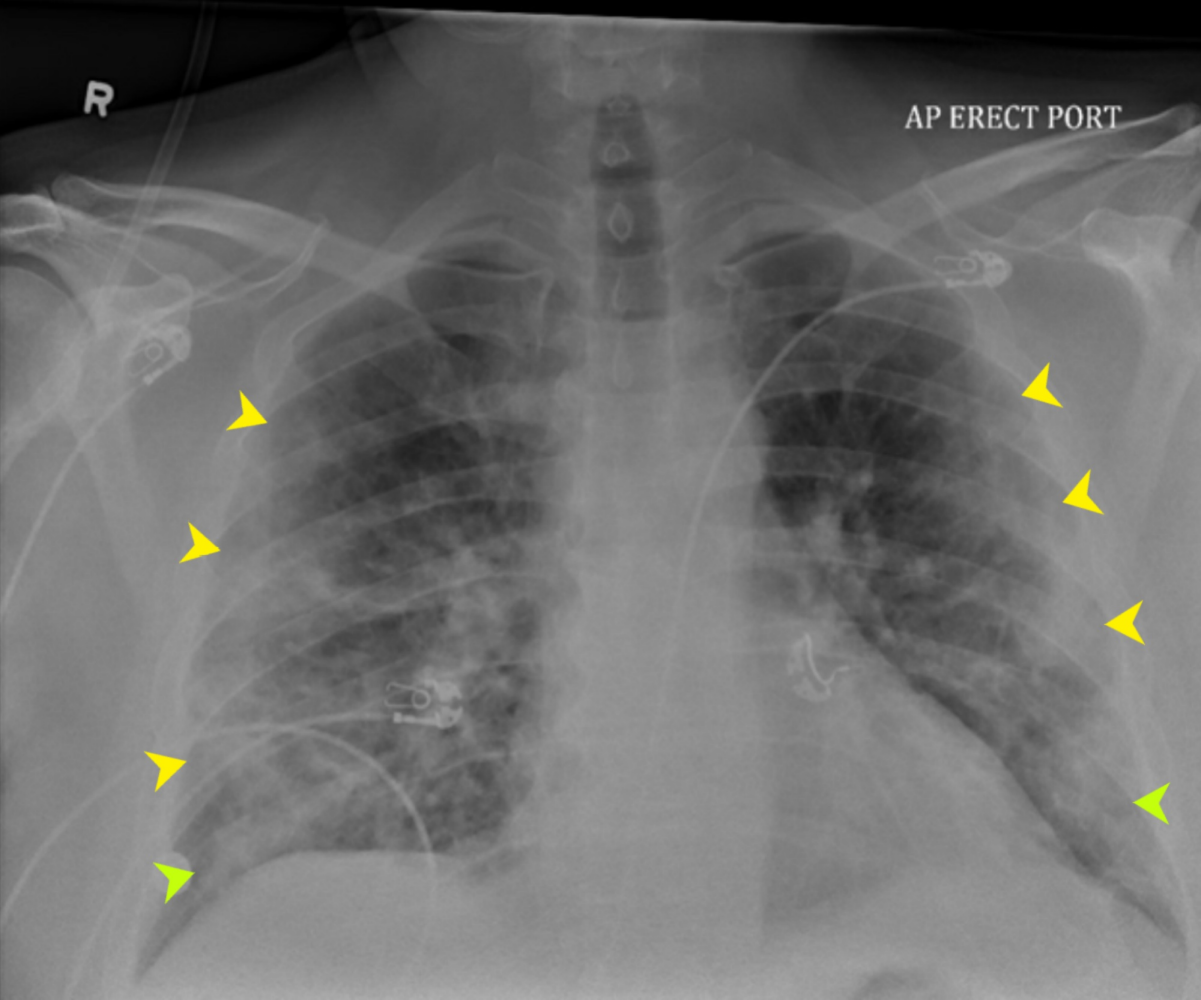
Chest X-ray shows bilateral, predominantly peripheral, pneumonic infiltrates most pronounced at the lung bases

Chest X-ray showed bilateral, predominantly peripheral and basal opacities (Figure 2).

The patient was admitted to the ICU for close monitoring. The next day, he started worsening clinically, was more hypoxic, with SpO_2_ 93% on six liters of oxygen via nasal cannula. Inflammatory markers were elevated (Table 2). Intubation was being considered and the patient was given one dose of TCZ 400 mg on the second day of admission. He remained stable on six liters of oxygen via nasal cannula and was managed conservatively. After two days in the ICU, he was transferred to the regular nursing floor. He was discharged from the hospital on the seventh day with home oxygen. On a one-month follow-up, the patient has been doing well.

Case 3

A 64-year-old male presented to the emergency room with complaints of fever and confusion for the last two days. His past medical history was significant for diabetes mellitus type 2, end-stage renal disease, and intermitted hemodialysis three times a week. He was tested for COVID-19 a day before admission. On admission, he had a fever of 103.1°F and was placed on two liters of oxygen via nasal cannula, SpO2 was 94%. He was otherwise hemodynamically stable and was admitted to the regular nursing floor. Laboratory results were as shown in Table 3.

**Table 3:** Clinical and Laboratory Parameters of Case 3 Temp, maximum recorded oral temperature; HR, maximum recorded heart rate; RR, maximum recorded respiratory rate; FiO_2_%, fraction of inspired oxygen; P/F ratio, ratio of arterial oxygen partial pressure (PaO_2_ in mmHg) to fractional inspired oxygen (expressed as percentage); WBC, serum white blood cell count; Abs Lym C, serum absolute lymphocyte count; Hb, serum hemoglobin; Hct, serum hematocrit; Plt count, serum platelet count; CRP, serum c-reactive protein; PT, prothrombin time; ALT, serum alanine aminotransferase; AST, serum aspartate aminotransferase; BUN, blood urea nitrogen; creat, serum creatinine; °F, degrees in Fahrenheit.

Parameters	Day 1	Day 2	Day 3	Day 4	Day 5	Day 6	Day 7	Day 8	Day 9	Day 10	Day 11
Temp (97.6°F-99.6°F)	103.1	104.4	102.2	102.6	102.8	100.7	99.5	98.7	98.4	98.1	98.1
HR (60-100/minute)	103	80	80	101	102	81	78	86	91	104	98
RR (12-20/minute)	21	23	18	22	24	16	20	20	20	20	18
FiO_2_ (%)	32	28	28	28	28	28	32	32	28	28	21
P/F ratio	-	-	-	-	-	-	195	-	-	352	-
WBC (3.70-11 x 10^3^/µL)	3.44	7.37	7.42	5.05	4.55	3.76	11.55	9.33	7.90	6.62	7.63
Abs Lym C (1.00-4.00 x 10^3^/µL)	0.54	0.81	1.08	0.51	0.64	0.44	0.81	0.28	0.73	0.53	1.13
Hb (11.5-15.5 gm/dL)	9.9	8.5	8.9	8.8	9.9	9.2	9.1	8.9	9.9	9.5	9.8
Hct (36.0%-46.0%)	29.6	26.2	26.2	26.7	29.8	28.0	27.8	27.2	29.1	28.8	29.4
Plt count (150-400 x 10^3^/µL)	125	118	104	117	146	143	165	193	248	261	287
CRP (0.0-1.0 mg/dL)	-	21.5	34.1	39	42.8	33.1	19.8	17.2	11.8	5.8	3.6
Ferritin (30.3-565.7 ng/mL)	-	2,709	3,840	6,456	11,020	10,767	10,298	10,447	9,503	7,805	6,042
Fibrinogen (200-400 mg/dL)	-	-	567	600	>860	803	717	659	686	610	538
Interleukin-6 (≤5 pg/mL)	-	-	141	99	-	-	-	-	-	-	-
ALT (5-50 U/L)	27	21	19	24	34	31	31	26	28	28	29
AST (7-40 U/L)	25	22	22	32	56	44	38	35	30	27	24
BUN (9-24 mg/dL)	43	48	66	79	45	68	91	54	85	45	79
Creat (0.73-1.22 mg/dL)	6.47	7.22	9.73	11.48	7.37	9.31	11.20	6.92	9.10	5.97	8.10

**Figure 3:**
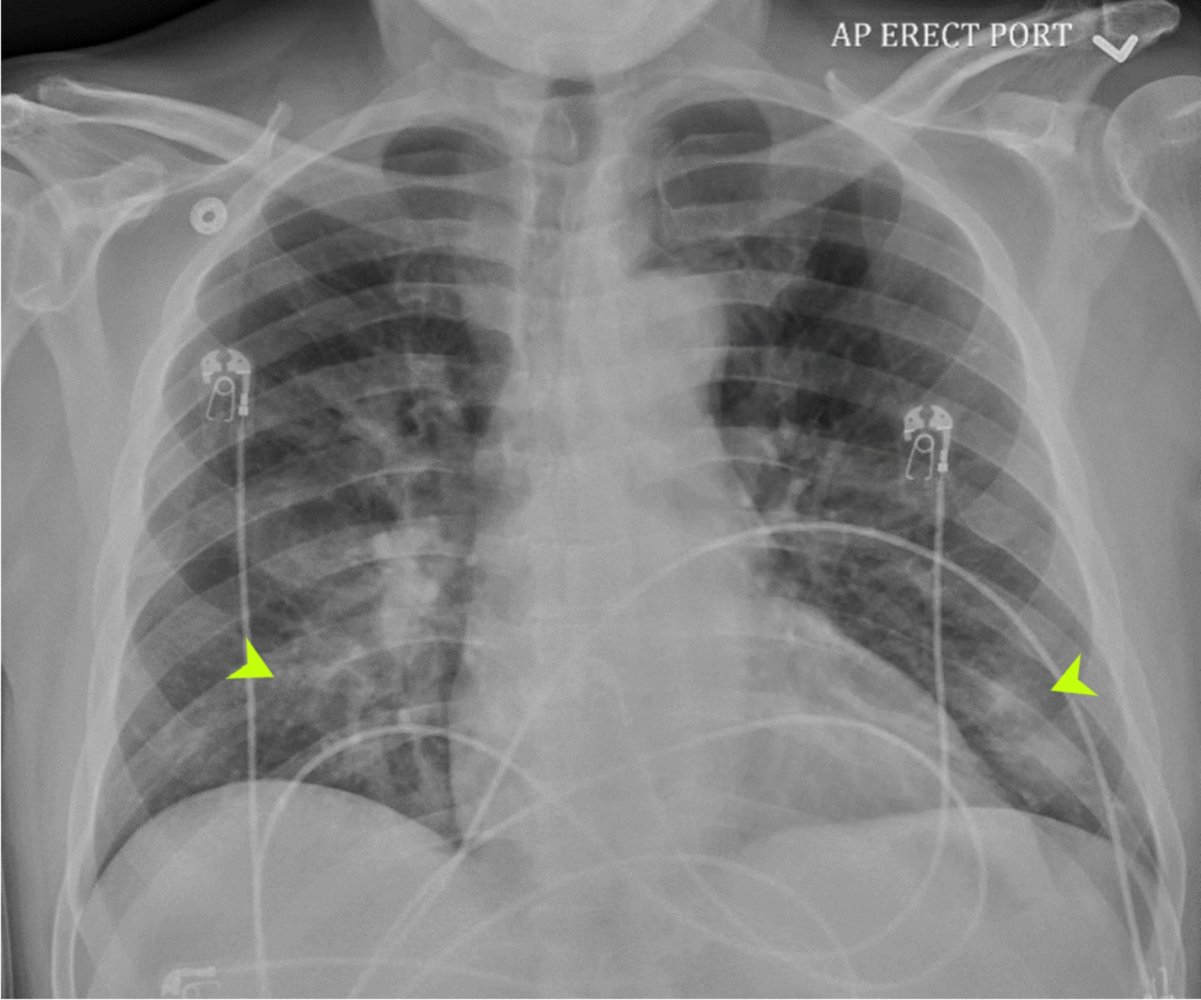
Chest X-ray shows patchy bibasilar infiltrate, right greater than left

A chest X-ray showed patchy bibasilar infiltrate, right greater than left (Figure 3).

He was started on azithromycin and hydroxychloroquine. Inflammatory markers trended upwards (Table 3). His oxygen requirements remained stable. He also continued to spike high-grade fevers. Given clinical worsening and uptrending inflammatory markers, he was given one dose of TCZ 400 mg on the fifth day of admission. His oxygen requirements went up on the seventh day of admission to up to four liters of oxygen via nasal cannula but improved subsequently. His inflammatory markers started trending down. He was taken off oxygen on the 10th day and discharged home on the 11th day of admission. On a one-month follow-up, the patient has been recovering well.

## Discussion

TCZ is an IL-6 inhibitor that is being used compassionately in patients with COVID-19. Studies have shown that patients with COVID-19, especially those with hypoxia and severe form of the disease, have elevated levels of IL-6, which likely is the reason for TCZ’s utility in these patients [2,3].

Multiple reports have been published regarding the use of TCZ in COVID-19 patients and have shown promising results. A study by Xu and colleagues evaluated 21 patients who were treated with TCZ and showed encouraging results. Of the 21 patients studied, four were reported as critical, although only one patient was reported to be on invasive ventilation. These patients had a wide range of IL-6 levels. All patients were shown to eventually recover and no deaths were reported [4]. Multiple case reports have also been published reporting the use of TCZ in COVID-19 patients with favorable outcomes [5-8]. Patients’ ages ranged from 42 to 60 years with one patient requiring mechanical ventilation.

A study by Luo and colleagues showed that TCZ helped decrease IL-6 levels, with clinical improvement in 10 out of 15 patients studied. Three of the patients died despite TCZ therapy. In that study, the pre-TCZ IL-6 levels of the patients who died were not significantly elevated (patients 1-3), and certain patients who clinically stabilized after TCZ had pre-TCZ IL-6 levels similar to those who died (patients 5, 6, 8-13). However, the study reported that the three patients who died were critically ill at the time of administration of TCZ [9]. The findings from this study suggest that the IL-6 inhibitor level by itself is not a reliable indicator of clinical worsening or improvement in COVID-19 patients. Furthermore, all three patients who died in this study were reported as critical, while other patients were clinically better, suggesting that early TCZ administration might have played a role in the clinical outcomes.

In our study, the three patients were aged 53 to 64 years. All three of them were deemed mild-moderately ill initially and received full doses of hydroxychloroquine (standard dosing of 400 mg twice a day followed by 200 mg twice a day for a total of five days) and azithromycin (standard dosing of 500 mg once a day for five days) except the first case. They were relatively doing well on admission but began to quickly deteriorate clinically, with inflammatory markers uptrending. The IL-6 levels were low on admission for cases 1 and 2, while for case 3 it had begun to downtrend. After the administration of TCZ, there was laboratory and clinical improvement in the patients’ conditions. The need for intubation and mechanical ventilation was avoided and they were soon discharged from the hospital.

Limitations of our study include small sample size, lack of control group, and publication bias. Further studies might help confirm our observation, in which case early TCZ administration might help improve patient morbidity and mortality and healthcare costs associated with ICU admission, mechanical ventilation, and prolonged hospital stay.

## Conclusions

Our experience with these patients suggests that early intervention is useful in COVID-19 patients. Patients who are found to be worsening based on clinical and/or laboratory parameters might benefit from the early administration of TCZ. Further studies are needed to shed more light on this area.
